# Progress Toward Measles Elimination — China, January 2013–June 2019

**DOI:** 10.15585/mmwr.mm6848a2

**Published:** 2019-12-06

**Authors:** Chao Ma, Lance Rodewald, Lixin Hao, Qiru Su, Yan Zhang, Ning Wen, Chunxiang Fan, Hong Yang, Huiming Luo, Huaqing Wang, James L. Goodson, Zundong Yin, Zijian Feng

**Affiliations:** ^1^National Immunization Program, Chinese Center for Disease Control and Prevention; ^2^World Health Organization Western Pacific Regional Office, Regional Reference Measles and Rubella Laboratory, National Institute for Viral Disease Control and Prevention, Chinese Center for Disease Control and Prevention; ^3^Global Immunization Division, Center for Global Health, CDC.

In 2005, the World Health Organization (WHO) Western Pacific Region countries, including China, resolved to eliminate measles by 2012 or as soon as feasible thereafter (*1*). As of 2018, nine* of the 37 Western Pacific Region countries or areas^†^ had eliminated^§^ measles. China’s Measles Elimination Action Plan 2006–2012 included strengthening routine immunization; conducting measles risk assessments, followed by supplementary immunization activities (SIAs) with measles-containing vaccine (MCV) at national and subnational levels; strengthening surveillance and laboratory capacity; and investigating and responding to measles outbreaks. Most recently, progress toward measles elimination in China was described in a 2014 report documenting measles elimination efforts in China during 2008–2012 and a resurgence in 2013 (*2*). This report describes progress toward measles elimination in China during January 2013–June 2019.^¶^ Measles incidence per million persons decreased from 20.4 in 2013 to 2.8 in 2018; reported measles-related deaths decreased from 32 in 2015 to one in 2018 and no deaths in 2019 through June. Measles elimination in China can be achieved through strengthening the immunization program’s existing strategy by ensuring sufficient vaccine supply; continuing to improve laboratory-supported surveillance, outbreak investigation and response; strengthening school entry vaccination record checks; vaccinating students who do not have documentation of receipt of 2 doses of measles-rubella vaccine; and vaccinating health care professionals and other adults at risk for measles.

## Immunization Activities

China introduced measles vaccine in 1965 and implemented nationwide measles vaccination in 1978 with the start of the national Expanded Program on Immunization (EPI). In 1986, the schedule was changed to include 2 MCV doses, with the first dose given at age 8 months and the second at age 7 years (the age of administration of the second dose was lowered to 18 months in 2005, as recommended in WHO guidelines).** Administrative coverage, calculated as the number of vaccine doses administered divided by estimated target population, is assessed monthly at the township level (the lowest administrative level), aggregated to the national level using vaccine administration and target population data reported by EPI clinics, and reported annually to WHO and the United Nations Children’s Fund (UNICEF). During 2013–2018, annual estimates of coverage with the first MCV dose (MCV1) and the second dose (MCV2) were both 99%. In 2016, among the 40,787 townships in China’s 31 mainland provinces, 40,089 (98%) reported >90% MCV2 coverage by age 3 years. In 2010, a nationwide SIA was conducted, during which 103 million children received MCV regardless of previous vaccination history. Each province then used a measles risk assessment tool developed by the Chinese Center for Disease Control and Prevention (China CDC) to determine the need for additional selective or nonselective follow-up SIAs in their jurisdiction. During 2013–2018, 56.9 million children and adults were vaccinated in these follow-up SIAs. During this time, the risk assessment–based SIA target population sizes decreased approximately sixfold, from 23 million in 2013 to 3 million in 2018. To ensure that school children are protected from vaccine-preventable diseases, China has had a national requirement since 2005 that vaccination status is checked upon entry to kindergarten and primary school; children with missing vaccine doses are referred to EPI clinics for catch-up vaccination. Although the school entry record check is required, receiving missing vaccine doses is not mandatory, and unvaccinated children are not excluded from school.

## Measles Surveillance Activities

Measles has been nationally notifiable since the 1950s, with aggregated data reported annually to the National Notifiable Disease Reporting System (NNDRS). In 1997, China developed a case-based, laboratory-supported measles surveillance system, initially in selected provinces and in parallel with NNDRS. The two surveillance systems were unified in 2009. Every suspected case is investigated by county-level China CDC staff members using a standardized, in-person questionnaire; outbreaks are investigated and reported by local China CDC staff members as needed. China’s Measles Laboratory Network comprises 31 provincial laboratories and one national laboratory that has been accredited by WHO as a Regional Reference Laboratory since 2003^††^ (*3*). Rubella case-based surveillance was integrated into the measles surveillance system in 2014. Since 2011, measles surveillance in China has met or exceeded WHO surveillance quality criteria (*4*).

## Measles Incidence and Epidemiologic Characteristics

From 2013 to 2014, measles incidence per million persons increased from 20.4 to 38.8; incidence subsequently declined each year, reaching 2.8 in 2018 (Table). Among confirmed cases reported during 2013–2018, the case count among infants aged <8 months (younger than the routinely recommended age for MCV1) decreased from 8,448 (31%) in 2013 to 532 (14%) in 2018 (Figure). Among the 1,839 measles cases reported in the first half of 2019, 109 (5.9%) were among infants aged <8 months, 965 (52.5%) were among children aged 8 months–14 years, and 765 (41.6%) were among persons aged ≥15 years. During 2013–2018, the number, size, and duration of measles outbreaks decreased steadily. Until 2019, almost all (98.9%) cases that had a measles virus genotype result were found to be the indigenous genotype H1. However, in the first half of 2019, this pattern changed: 82% of genotyped measles viruses were found to be import-associated genotypes B3 or D8 (Table) (*5*).

**TABLE:** Epidemiologic characteristics of reported measles, cases, outbreaks, and isolate genotypes — China, January 2013–June 2019

Characteristic	Year						
	2013	2014	2015	2016	2017	2018	Jan–Jun 2019
Measles incidence, cases per million population*	20.42	38.84	31.09	18.11	4.31	2.84	1.27
No. of 31 total provinces with incidence <1 per million population	1	0	0	2	4	5	NA
No. of measles cases	27,646	52,628	42,361	24,820	5,941	3,940	1,839
**Age group distribution, no. (%)**							
<8 mos	8,448 (30.6)	11,193 (21.3)	10,575 (24.9)	4,652 (18.7)	950 (16.0)	542 (13.8)	109 (5.9)
8–23 mos	8,227 (29.8)	11,928 (22.7)	10,070 (23.8)	5,910 (23.8)	1,786 (30.0)	1,231 (31.2)	530 (28.8)
2–6 yrs	2,890 (10.4)	4,554 (8.6)	3,933 (9.3)	2,521 (10.2)	866 (14.6)	554 (14.1)	233 (12.7)
7–14 yrs	648 (2.3)	1,696 (3.2)	1,313 (3.1)	971 (3.9)	445 (7.5)	273 (6.9)	202 (11)
≥15 yrs	7,433 (26.9)	23,257 (44.2)	16,470 (38.9)	10,766 (43.4)	1,894 (31.9)	1,340 (34.0)	765 (41.6)
**No. of vaccine doses received by measles patients aged 8 mos–14 yrs^†^**							
0	7,636 (64.9)	10,964 (60.3)	9,158 (59.8)	5,332 (56.7)	1,146 (37.0)	629 (30.5)	127 (14.6)
1	1,889 (16.1)	2,947 (16.2)	2,725 (17.8)	1,865 (19.8)	945 (30.5)	749 (36.4)	311 (35.9)
≥2	724 (6.1)	1,577 (8.7)	1,453 (9.5)	1,128 (12.0)	495 (16.0)	551 (26.8)	340 (39.2)
Unknown	1,516 (12.9)	2,690 (14.8)	1,980 (12.9)	1,077 (11.5)	511 (16.5)	129 (6.3)	89 (10.3)
Laboratory-confirmed (%)	96.3	96.3	96.3	96.1	85.6	96.5	92.6
Male sex (%)	59.8	56.5	56.2	55.2	57.2	57.6	56.5
No. of measles-related deaths	24	28	32	18	5	1	0
Measles deaths per million population	0.018	0.020	0.023	0.013	0.004	0.001	0
Administrative MCV2 coverage (%)	99.6	99.9	99.4	99.4	99.4	99.2	NA
No. of persons vaccinated in SIAs (million)	22.67	12.81	9.12	4.06	5.44	2.84	NA
No. of outbreaks reported^§^	109	283	329	230	38	37	18
No. of outbreak-related cases	436	2,080	1,847	1,235	238	158	83
Median no. of cases per outbreak (range)	2 (2–29)	3 (2–271)	2 (2–278)	4 (2–122)	3 (2–59)	3 (2–29)	3 (2–14)
Median outbreak duration, days (range)	8 (1–44)	7 (1–158)	8 (1–245)	85 (1–65)	13 (1–44)	11 (1–28)	9 (1–35)
Measles virus genotypes (no. identified)^¶^	H1 (2,208); B3 (3); D8 (51); D9 (47)	H1 (4,872); B3 (10); D8 (3); D9 (9); G3 (1)	H1 (3,948); D9 (1)	H1 (2,467); D8 (3)	H1 (400); B3 (1); D8 (10)	H1 (155); B3 (3); D8 (8)	H1 (24); B3 (18); D8 (91)

**Abbreviations:** MCV = measles-containing vaccine; MCV2 = second dose of MCV; NA = not available; SIA = supplementary immunization activity.

* Incidence for January–June 2019 is annualized.

^†^ No. of doses of MCV received by patient as of date of measles illness onset.

^§^ In China, a measles outbreak is defined as the occurrence, within a 10-day period, of either two or more confirmed measles cases in a village, district, school, or similar unit or five or more confirmed measles cases in a township.

^¶^ https://journals.plos.org/plosone/article?id=10.1371/journal.pone.0218782.

**FIGURE:**
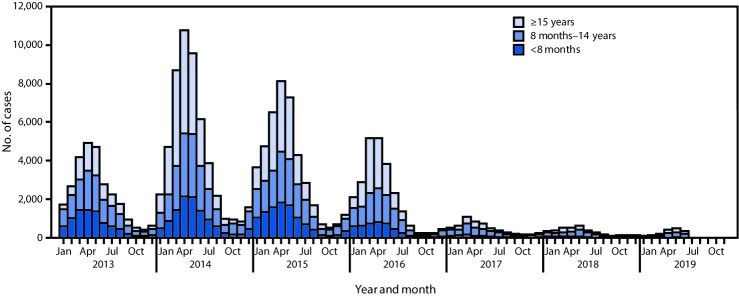
Confirmed measles cases,* by age group — China, January 2013–June 2019 * Confirmed cases include those that are laboratory-confirmed, epidemiologically linked to a laboratory-confirmed case, or clinically compatible.

## Discussion

Progress toward measles elimination in China has been considerable. Measles cases, incidence, and outbreaks were all at historically low levels in 2017 and 2018 and have decreased further through June 2019. Measles deaths are now rare in this country of 1.4 billion persons, with just one measles-associated death reported in the last 18 months.

Laboratory-supported surveillance is critical for guiding measles elimination activities and strengthening routine immunization. Outbreak investigations have identified gaps in population immunity that are addressed with follow-up immunization activities and program strengthening. The risk assessment–based SIA target population sizes markedly decreased during 2013–2018, providing indirect evidence of strengthened routine immunization service delivery.

Consultations with international partners, including CDC, WHO, UNICEF, the World Bank, the Japan International Cooperation Agency, and the Measles & Rubella Initiative^§§^ have helped guide activities. Research and evaluation have also provided valuable information for measles elimination. MCVs used in China were found to be highly immunogenic in infants aged 8 months, and coadministration of Japanese encephalitis vaccine did not reduce measles seroconversion rates (*6*). In a Chinese study of risk factors for measles in children aged 8 months–14 years after a nationwide SIA, the estimated measles vaccine effectiveness among children was >95%, and being unvaccinated was the leading risk factor for infection (*7*). In addition, hospitals were important sites of measles virus transmission, and internal migration was associated with risk for measles acquisition (*7*). In a 2013 assessment of vaccination coverage in China during an outbreak following a nationwide SIA, administrative vaccination coverage might have overestimated coverage by 5%–10% (*8*). Finally, application of false contraindications to vaccination led to missed opportunities to immunize some children against measles (*9*).

Research and evaluation have led to action. In 2015, the Chinese Ministry of Health recommended measles vaccination for hospital professionals, and in 2017, China CDC and WHO hosted an international consultation to improve coverage assessment methods. Immunogenicity results provided evidence of adequate seroconversion when MCV1 is given at age 8 months, satisfying the WHO evidence requirement for routine MCV1 administration before age 9 months. EPI clinics are now directed to vaccinate migrant children after 3 months of residence.

Mathematical modeling has also proven useful. A metapopulation measles virus transmission model that estimated the basic reproduction number for measles to be 18 nationwide indicated that by 2014, the effective reproduction number was 2.3 and was <1 in 14 provinces (*10*). The model predicts that measles will eventually be eliminated by the current strategy and that measles elimination can be accelerated by vaccinating middle school and high school students lacking evidence of receipt of 2 MCV doses.

The global nature of measles virus transmission is evident in the patterns of measles virus importations and exportations. China’s measles surveillance system detects imported cases, and other countries have detected importations from China. For example, during January 2016–June 2019, CDC detected only one importation from China into the United States, compared with six, four, and five such importations each year during 2013–2015, respectively, supporting the understanding that cooperation among countries in fighting measles can benefit all countries.

The findings in this report are subject to at least two limitations. First, administrative coverage can be affected by inaccurate population estimates leading to under- or overestimates of coverage (*8*). Second, despite meeting WHO Western Pacific Region surveillance quality indicators, surveillance might underestimate incidence because not all measles patients come to medical attention, and some medically attended cases might not be reported.

China is approaching measles elimination, but the high transmissibility of measles virus, the size and density of China’s population, and the persistence of global measles virus transmission mean that measles will continue to be detected in China for years to come. Elimination can be achieved with an updated action plan that includes ensuring sufficient vaccine supply, continuing to improve laboratory-supported surveillance and outbreak response, strengthening the school-entry vaccination record check, vaccinating students lacking documentation of receipt of at least 2 doses of measles/rubella vaccine, and vaccinating health care professionals and other adults at risk for measles. Data sharing and cooperation among countries and international organizations will continue to be critically important in the global effort to eliminate and eventually eradicate measles.

**Summary**

**What is already known about this topic?**

China has historically had high measles incidence and many associated deaths. A comprehensive measles elimination plan during 2006–2012 substantially reduced measles incidence; however, a resurgence occurred during 2013–2015.

**What is added by this report?**

In China, measles surveillance, outbreak response, research, and program evaluation were used to strengthen routine immunization and target immunization activities for eliminating measles. Measles incidence declined from 31 per million in 2015 to 2.8 in 2018; only one measles-associated death has been reported during 2018–June 2019.

**What are the implications for public health practice?**

The World Health Organization–recommended strategy to eliminate measles can be effective, including in large, densely populated countries like China.

## Footnotes

* Australia, Brunei, Cambodia, Hong Kong (China), Macao (China), Japan, New Zealand, South Korea, and Singapore.

† The Western Pacific Region, one of the six regions of WHO, consists of 37 countries and areas with a population of almost 1.9 billion, including American Samoa (USA), Australia, Brunei, Cambodia, China, Cook Islands, Federated States of Micronesia, Fiji, French Polynesia (France), Guam (USA), Hong Kong (China), Japan, Kiribati, Laos, Macao (China), Malaysia, Marshall Islands, Mongolia, Nauru, New Caledonia (France), New Zealand, Niue, Northern Mariana Islands (USA), Palau, Papua New Guinea, Philippines, Pitcairn Islands (UK), Samoa, Singapore, Solomon Islands, South Korea, Tokelau (New Zealand), Tonga, Tuvalu, Vanuatu, Vietnam, and Wallis and Futuna (France).

§ Measles elimination is defined as the absence of endemic measles virus transmission in a defined geographical area (e.g., region or country) for ≥12 months with a well-performing surveillance system.

¶ Population of 1.4 billion, not including Hong Kong Special Administrative Region, Macao Special Administrative Region, and Taiwan.

** https://www.who.int/immunization/documents/positionpapers/en/.

†† https://www.who.int/immunization/monitoring_surveillance/burden/laboratory/measles/en/.

§§ The Measles & Rubella Initiative is a partnership established in 2001 as the Measles Initiative, spearheaded by the American Red Cross, CDC, the United Nations Foundation, UNICEF, and WHO. https://measlesrubellainitiative.org/.
